# Analysis of myeloid neoplasms with isolated trisomy 19 reveals a novel MDS subgroup characterized by the presence of ring sideroblasts, fibrosis and *SRSF2* and/or *ASXL1* mutations

**DOI:** 10.1007/s12308-025-00659-1

**Published:** 2025-10-09

**Authors:** Konnie M. Hebeda, Ludmila Boudová, Maarten F. Corsten, Nikola Ptáková, Torsten Haferlach, Aniek O. de Graaf, Jaroslav Cermak, Tomas Vanecek, Joop H. Jansen, Marian J. P. L. Stevens-Kroef, Leonie I. Kroeze

**Affiliations:** 1 Department of Pathology, Radboud University Medical Center, Nijmegen, Netherlands; 2 Department of Pathology, Medical Faculty, Charles University, Pilsen, Czech Republic; 3 Biopticka Laborator, Pilsen, Czech Republic; 4 Internal Medicine, Meander Medisch Centrum, Amersfoort, Netherlands; 5 Department of Biology and Medical Genetics, 2nd Faculty of Medicine, University Hospital Motol, Prague, Czech Republic; 6 Munich Leukemia Laboratory, Munich, Germany; 7 Laboratory Hematology, Department of Laboratory Medicine, Radboud University Medical Center, Nijmegen, Netherlands; 8 Institute of Hematology and Blood Transfusion, MDS Registry, Prague, Czech Republic; 9 Department of Human Genetics, Radboud University Medical Center, Nijmegen, Netherlands

**Keywords:** Myelodysplastic syndrome, Classification, Trisomy, Ring sideroblast, Mutation

## Abstract

We collected 97 cases of myeloid neoplasia with the rare cytogenetic event of isolated trisomy 19 (+19), with the aim to characterize this group clinically and pathologically. 51 patients with myelodysplastic syndrome (MDS +19) and 11 patients with myelodysplastic/myeloproliferative neoplasms (MDS/MPN +19) presented with +19 at disease onset and were further analyzed. Patients with insufficient data were excluded. We collected additional clinical and laboratory data and performed mutation analysis on available bone marrow biopsies. The 62 patients of both disease groups turned out to be remarkably homogeneous in terms of male sex (85%), the presence of anemia with increased numbers of ring sideroblasts (RS, 80%), the absence of an *SF3B1* mutation (95%), and the overall rather consistent presence of *SRSF2* (61%) or *ASXL1* (39%) mutations. MDS +19 patients with available follow-up (1 month to 7.5 years) presented or progressed with significant fibrosis (45%), leuko- or monocytosis (13%) or acute leukemia (28%). Compared to a control cohort of 23 patients with MDS and an *SRSF2* mutation, but without isolated +19 (MDS-*SRSF2*), the 16 MDS +19 patients with *SRSF2* mutation and the 12 MDS +19 patients with an *ASXL1* mutation showed a striking difference in the presence of ≥ 15% RS (73% and 67% versus 17% in MDS-*SRSF2*) and the occurrence of fibrosis (44% and 57% versus 4% in MDS-*SRSF2)*. Although all individual features observed in the MDS +19 and MDS/MPN +19 cohorts are seen in MDS and MDS/MPN in general, their combination is rather unique and provides clues regarding disease evolution in this rare, cytogenetically defined group of myeloid neoplasia.

**Supplementary Information:**

The online version contains supplementary material available at 10.1007/s12308-025-00659-1.

## Introduction

Trisomy 19 (+19) is a well-known cytogenetic aberration in myeloid neoplasms, mostly combined with other structural abnormalities and frequently acquired as a secondary event during disease progression, especially in secondary acute myeloid leukemia (AML), pediatric AML, acute megakaryoblastic leukemia, and chronic myeloid leukemia [1–4]. It is, however, rare as an isolated event, occurring in 0.4% of patients with myelodysplastic syndrome (MDS), in 1% of AML and 0.02% of chronic myelomonocytic leukemia patients (CMML) [1, 5–7]. As a sole cytogenetic abnormality in MDS, +19 corresponds to an intermediate cytogenetic risk according to IPSS-R, with 25% of the patients predicted to progress to AML within 2.5 years and a median survival of 2.7 years [5, 8]. The molecular background of MDS +19 and MDS/MPN +19 is hitherto unknown.

## Methods

### Data collection

We searched the datafiles of the 5 Dutch cytogenetic centers (Nijmegen, Rotterdam, Amsterdam, Leiden, Groningen) and 2 hospitals in the Czech Republic (Pilsen and Prague) for patients with myeloid neoplasia (MN) and isolated +19. Subsequently additional information, including clinical and molecular reports, was requested from the original hematology departments in the Netherlands and the Czech Republic. Trephine biopsy slides were requested from the pathology departments for examination. To confirm the unexpected recurrent combination of an *SRSF2* mutation with increased ring sideroblasts (RS), but lack of *SF3B1* mutation, we enriched the series with diagnostic and molecular information of patients with myeloid neoplasia with isolated +19 from the Munich Leukemia laboratory (MLL, Germany). No clinical information was available for these cases.

A control cohort of 23 bone marrow biopsies of MDS patients with an *SRSF2* mutation (MDS-*SRSF2*) without isolated +19, was identified from the archive of Radboudumc. Data on diagnosis, anemia at presentation, presence of RS, histology, and cytogenetics were collected. No treatment or follow-up data were available for this cohort. Finally, a comparison with different MDS subgroups from literature was performed.

### Review of trephine biopsies

The original bone marrow (BM) biopsy slides (including H&E, PAS and reticulin stain and immunohistochemistry for CD34) and the tissue blocks were received from pathology laboratories in the Netherlands and the Czech Republic. Aspirate smears were not available for review. The BM biopsies of the 23 control MDS-*SRSF2* patients had all been analyzed at the Radboudumc. For the MLL patients the reports of the BM aspirates but no biopsies were available, therefore information on fibrosis is lacking.

### Mutation analysis on tissue blocks

In total, we obtained mutation analysis data of the peripheral blood and/or bone marrow of 55 of the 97 patients: either from the clinical files (n = 11), the MLL database (n = 18) or by performing NGS on BM paraffin blocks, either in Pilsen (n = 26) or Nijmegen (n = 4, nr. 22, 27, 94, 95). 4 analyses on blocks of biopsies between 2000 and 2015 failed due to degraded DNA. DNA of the decalcified and paraffin embedded BM biopsies was extracted from tissue slides using an FFPE DNA kit (automated on Maxwell RSC 48 Instrument, Promega, Madison, Wisconsin, USA) or a DNA/RNA Kit (Qiagen). The DNA was purified using AMPure XP Beads (Beckman Coulter, Brea, CA, USA). Purified DNA was quantified using the Qubit Broad Range DNA Assay (Thermo Fisher Scientific, Waltham, Massachusetts, USA). Mutation analysis was performed with an NGS panel of genes relevant for myeloid malignancies. Either the Variant Plex Core Myeloid kit (Archer DX, Boulder, CO, USA, 37 gene targets) and the Fusion Plex Myeloid kit (Archer DX, Boulder, CO, USA, both fusion and mutations of 84 gene targets) on NextSeq500 platform (Pilsen), or a custom 27 or 36‐gene panel of single‐molecule‐tagged molecular inversion probes (smMIPs, Nijmegen [9]) on NovaSeq6000 platform (Illumina) (see supplementary Table 2 for the gene panels) were used. Variants were called using ≥ 2% VAF and a minimum of 10 variant reads (smMIPs) or ≥ 5% VAF and 50 variant reads (Archer).

## Results

### Clinical data

**Fig. 1:**
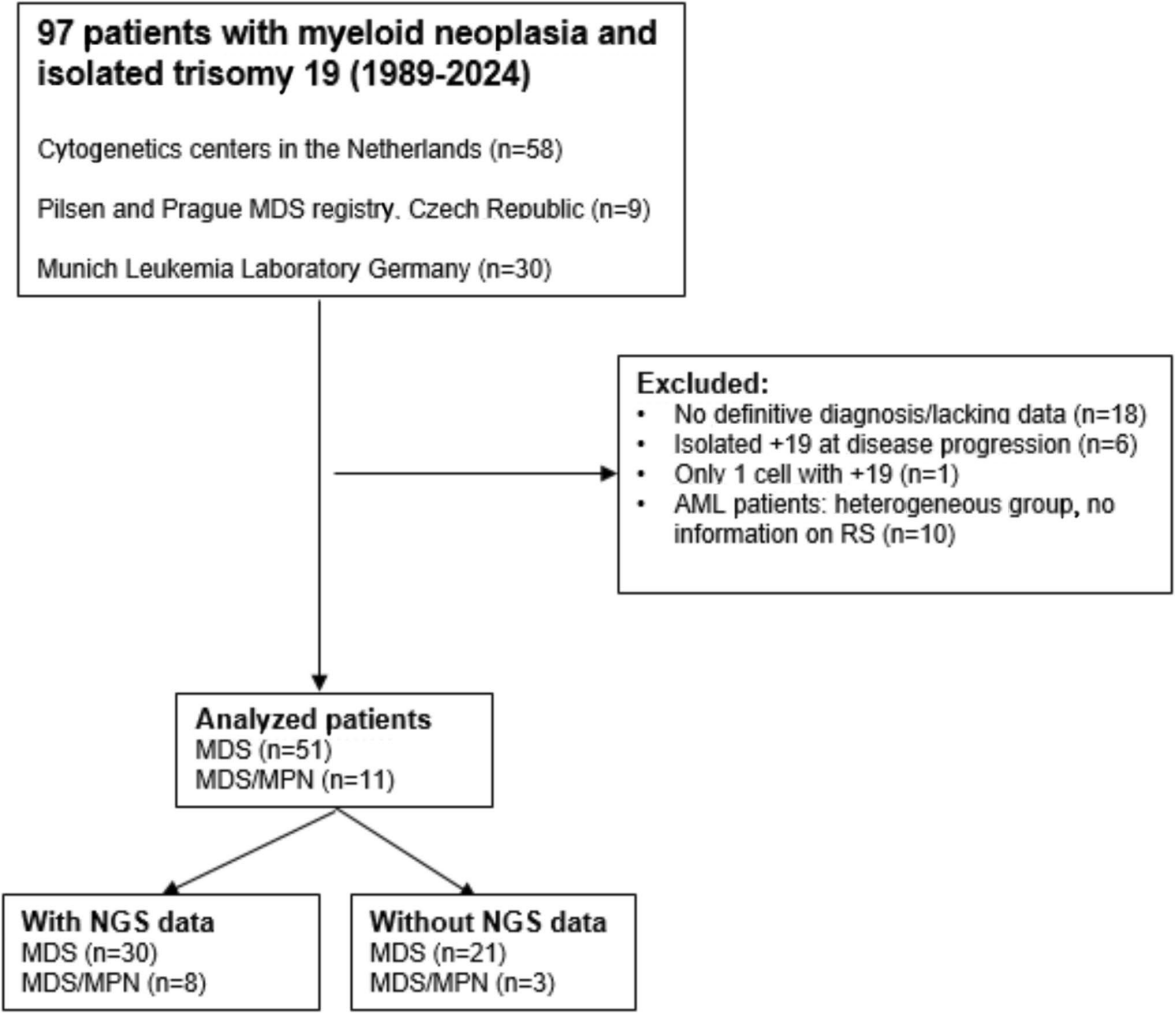
Overview of patients with myeloid neoplasia and isolated +19 from 3 cohorts, case selection for further analysis and availability of NGS data. Data for each patient are provided in supplementary Table 1

We identified a total of 97 patients with MN and isolated + 19, diagnosed between 1989 and 2024. Data on all patients are summarized in supplementary Table 1. Of the 97 patients, 25 were excluded due to an unspecified MN or a previous MN without + 19 (see Fig. 1 for the case selection). 10 patients with AML were excluded because of lacking clinical and pathological data in this small group. We did not identify any patients with an isolated myeloproliferative neoplasm and isolated + 19.

Eventually 51 patients with MDS (MDS +19) and 11 patients with MDS/MPN (MDS/MPN +19) were further analyzed (total cohort n = 62). Given that cases were collected over a 35-year time span, different classification systems were used for original diagnoses. Therefore, we re-classified cases based on the WHO5 [10] and ICC [11] classification systems (see Table 1). The MDS/MPN +19 patients consisted of 4 CMML and 1 atypical CML, 6 could not be further classified.

**Table 1:** Summary of characteristics of myeloid neoplasia patients with isolated +19

ICC 2022; WHO 2022	N	Male/total (%)	*ASXL1* mutation	*SRSF2* mutation	> 15% ring sideroblasts
**All patients**	**62**	**53/62 (85%)**	**15/38 (39%)**	**23/38 (61%)**	**32/40 (80%)**
**MDS**	**51**	**42/51 (82%)**	**12/30 (40%)**	**16/30 (53%)**	**28/35 (80%)**
MDS, NOS; MDS-LB	34	28/34 (82%)	8/20	12/20	22/24
MDS-SF3B1	1	1/1 (100%)	0/1	0/1	1/1
MDS-EB; MDS-IB1	6	4/6 (67%)	0/2	2/2	3/4
MDS/AML; MDS-IB2	10	9/10 (90%)	4/7	2/7	2/6
**MDS/MPN**	**11**	**11/11 (100%)**	**3/8 (38%)**	**7/8 (88%)**	**4/5 (80%)**
CMML	4	4/4 (100%)	1/2	2/2	0/0
other MDS/MPN	7	7/7 (100%)	2/6	5/6	4/5

The MDS +19 and MDS/MPN +19 patients were predominantly male (82% and 100%, respectively) with a median age of 75 years for MDS +19 (range 58–91), and 73 years for MDS/MPN +19 (range 52–84). Partial clinical data was available for 37 of the 62 patients. All MDS +19 patients presented with anemia, less frequently accompanied by leukocytopenia (41%) and/or thrombocytopenia (44%). In 6 MDS +19 patients, splenomegaly was reported at disease onset (data not shown).

The 6 MDS/MPN +19 patients with available information showed anemia (67%), thrombocytopenia (50%), leukocytosis (50%), monocytosis (33%) and/or thrombocytosis (17%) (suppl. Table 1).

The data of the control cohort of 23 MDS-*SRSF2* patients are described in Table 2. The majority consisted of male patients (74%), with a median age of 69 years (range 47–82) and anemia at presentation in 91%. Trisomy 19 was present in one of the control patients with ≥ 15% RS, although not isolated but combined with deletion 7q. Clinical data of other MDS subgroups from literature are summarized in Table 2.

**Table 2:** Study cohort of MDS patients with isolated +19 and control MDS patients with *SRSF2* mutation, compared to MDS patients from the literature

	MDS +19 (n= 51)	MDS +19 with *SRSF2* mutation (*n* = 16)	MDS +19 with *ASXL1* mutation (*n* = 12)	MDS-*SRSF2* mutation (*n* = 23)	All MDS	MDS +19* (n*= 21)	All MDS-RS	MDS-*SF3B1* mutation	MDS-*SRSF2* mutation
	This study				Literature				
Trisomy 19	100%	100%	100%	2%	0.4–1%	100%	n.a	0%	n.a
Median age	75 yrs	75 yrs	74 yrs	69 yrs	69–76 yrs	74 yrs	66–74 yrs	70–77 yrs	70–75 yrs
Male sex	82%	75%	92%	74%	55–70%	80%	49–60%	50–64%	64–94%
Anemia	100%	100%	100%	91%	54%	100% (13/13)	100%	57–100%	n.a
Low blasts (no EB/IB)	69%	75%	58%	74%	57–70%	74% (14/19)	74–85%	92–100%	27–43%
RS ≥ 15%	78%	73%	67%	17%	10–21%	38% (5/13)	100%	63–75%	6–12%
*SF3B1* mutation	3%	0%	0%	4%	11–33%	n.a	47–81%	100%	1–2%
*SRSF2* mutation	53%	100%	17%	100%	3–20%	n.a	< 2–15%	0–3%	100%
*ASXL1* mutation	40%	13% (2/16)	100%	39%	12–27%	n.a	20–22%	0–17%	47–58%
*SRSF2-ASXL1* co-mutation	4%	13%	17%	39%	0.7–2%	n.a	20%	0%	58%
Fibrosis onset + FU	45%	44% (4/9)	57% (4/7)	4% (1/23)	1.4–17%	n.a	n.a	1%	2%
					[5, 8, 12–23]	[24–40]	[12–15, 17, 21, 41–46]	[13, 15, 22, 43, 46–48]	[13–15, 20, 22, 43]

EB/IB: excess/increased blasts; FU: follow-up; RS: ring sideroblasts; yrs: years

### Bone marrow reports and histology review

**Fig. 2:**
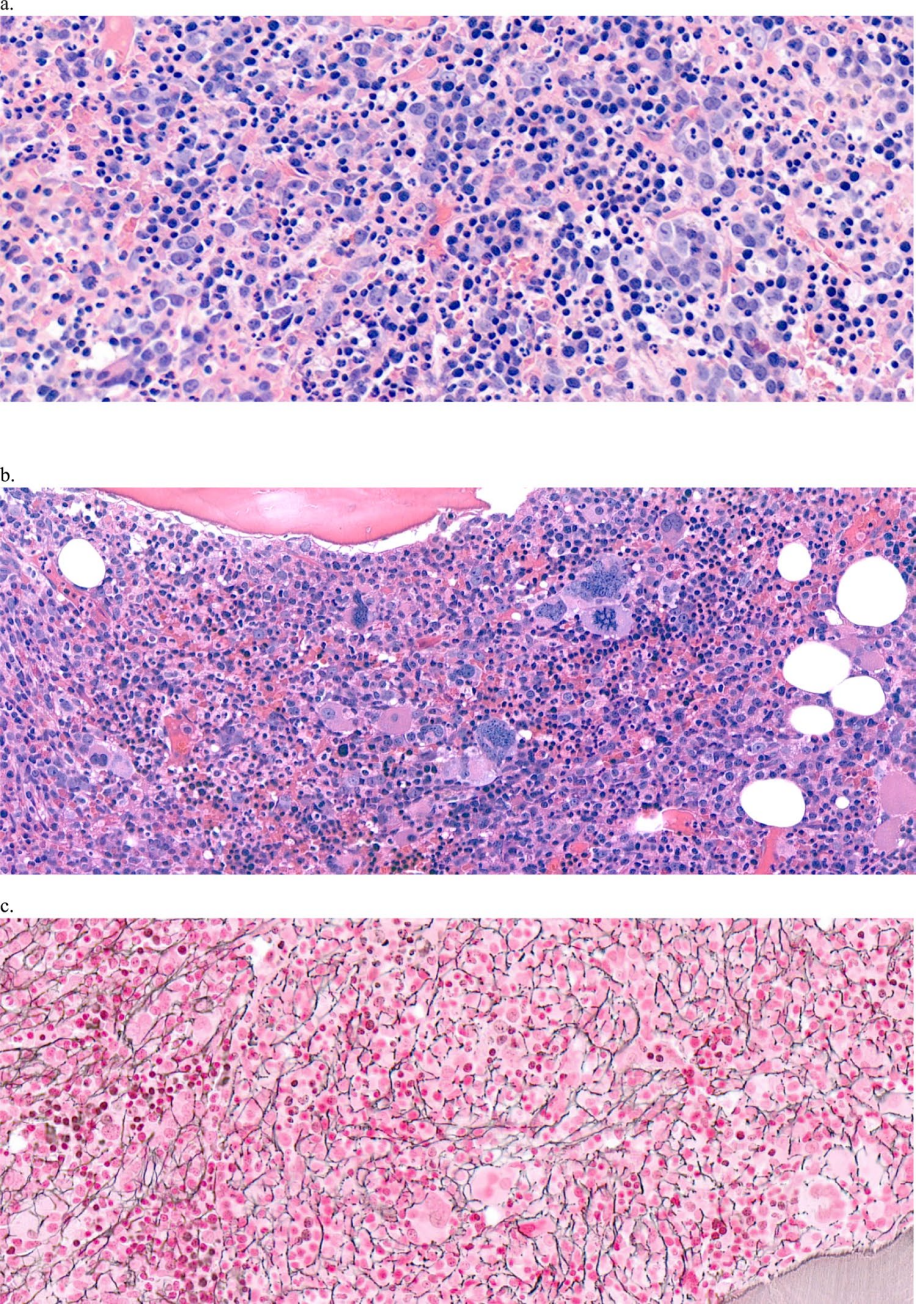
Histology of bone marrow biopsies of MDS +19 and MDS/MPN +19. **a.** Example of MDS +19 with erythroid hyperplasia and dysplasia (patient 9), H&E stain, 40x. **b** and **c**. Example of CMML +19 with myeloid hyperplasia, dysplastic (small hypolobated nucleus) or myeloproliferative (enlarged hyperlobated nucleus) aspect of the megakaryocytes and diffuse fibrosis grade 2 (patient 60), **b**. H&E stain and c. reticulin stain, 35x

We were able to review the original trephine biopsy slides of 26 MDS +19 and 7 MDS/MPN +19 patients, and follow-up slides of 13 patients. In 6 patients no BM biopsy had been performed. Combined with additional reports of BM biopsies and BM aspirates of MLL patients, data on the morphology were available for 86% of MDS +19 and 91% of MDS/MPN +19 patients. At disease onset low blast counts in blood and bone marrow were reported in 69% of MDS +19 patients and in 100% of the MDS/MPN +19 patients (data available in 8/11). Of the patients with reported RS numbers, 80% of both MDS +19 and MDS/MPN +19 had ≥ 15% RS, in the range 15–89% RS. In 91% of MDS +19 and all MDS/MPN +19 patients the marrow was hypercellular (data not shown). While erythroid hyperplasia (myeloid/erythroid ratio ≤ 1) was seen in 57% of MDS +19 patients (see example patient 9 in Fig. 2a), MDS/MPN +19 was characterized by myeloid hyperplasia (78%, see example patient 60 in Fig. 2b), except for 2 patients with MDS/MPN, NOS. Significant fibrosis (grade 2 or 3) was present at MDS +19 onset in 17% of the patients and increased to 45% of the patients during follow-up. In MDS/MPN + 19, one patient with CMML presented with fibrosis (patient 60 in Fig. 2b and 2c), 2 others developed fibrosis during follow-up (27%).

The bone marrow of the 23 control MDS-*SRSF2* patients was hypercellular in 87%, with erythroid hyperplasia in 26% and low blasts in 74%. Only 4 patients (including 1 MDS-*SF3B1* patient) had RS ≥ 15%. No significant fibrosis (grade 2 or 3) was present at disease onset, 1 patient developed fibrosis grade 2 during follow-up.

### Molecular analysis

Altogether, mutation data were available for 59% (30/51) of the MDS +19 and 73% (8/11) of the MDS/MPN +19 patients (see results in Table 1). Recurrently mutated genes (in > 2 of these patients) were *SRSF2* (61%), *ASXL1* (39%), *TET2* (24%), *U2AF1* (18%), *SETBP1* (16%), *RUNX1* (11%), and *KIT* (8%). Mutations in either *SRFS2* and/or ASXL1 were detected in 87% of MDS +19 and in all 8 MDS/MPN +19 patients. *ASXL1* and *SRSF2* mutations co-occurred in 4 patients. In 40% of cases with an *ASXL1* mutation this was combined with an *U2AF1* mutation, while *U2AF1* was only reported once without *ASXL1*. An *SF3B1* mutation was present in only 2 cases: one as a single mutation and one combined with *SRSF2* and 2 *ASXL1* mutations. No mutations were detected in the splicing factor *ZRSR2*.

A separate analysis of MDS +19 patients with an *SRSF2* mutation (MDS +19-*SRSF2*, 53%) or an *ASXL1* mutation (MDS +19-*ASXL1*, 40%) is presented in Table 2. The characteristics of both groups were comparable to the whole MDS +19 cohort.

In the MDS-*SRSF2* control cohort 2 patients had an *SRSF2* mutation only, whereas the other cases showed additional mutations. *ASXL1* was co-mutated in 9 (39%) and *SF3B1* in one of the cases. Other recurrently mutated genes were *RUNX1* (43%), *TET2* (35%), *IDH1* (17%), *IDH2* (13%) and *TP53* (13%). Characteristics of our study cohorts were compared to patients with MDS and MDS+19, MDS-RS, MDS-*SRSF2* and MDS-*SF3B1* from the literature (Table 2).

### Treatment and follow-up

Treatment data could be obtained for 20 MDS +19 and 2 MDS/MPN +19 patients, and follow-up data (ranging from 1 month to 7.5 years) for 36 patients. Patients with MDS +19 with low blast counts generally received supportive treatment, including blood transfusions and growth factors. MDS +19 patients with available follow-up progressed with leuko- or monocytosis (13%) and/or increase of blasts (44%), resulting in 9 acute leukemias (28%, 8 myeloid, 1 T-cell phenotype). In these patients azacytidine or hydroxyurea treatment was added. Only 2 MDS +19 and 1 MDS/MPN +19 patient received an allogeneic bone marrow transplant. Supplementary Fig. 1 shows a Kaplan–Meier curve for MDS +19 patients, with a 5-year survival rate of 30%. Due to the low number of MDS/MPN +19 patients, no survival analysis has been performed for this group.

## Discussion

The MDS +19 and MDS/MPN +19 patients turned out to be remarkably homogeneous in terms of presence of increased RS, lack of *SF3B1* mutation, the presence or development of fibrosis during follow-up, and presence of *SRSF2* or *ASXL1* mutations, although not all clinical and pathological information could be retrieved. The disease presentation for MDS +19 patients with an *SRSF2* mutation and patients with an *ASXL1* mutation was similar (Table 2). *SRSF2* and *ASXL1* mutations are quite common in MDS (10–20% and 17%, respectively) and MDS/MPN (35% and 50–90%, respectively) in general, where they are predictive for adverse outcome despite low blast counts. However, *SRSF2* and *ASXL1* mutations are less common in combination with RS (*SRSF2* < 2–12%, *ASXL1* < 22%) [12–15, 41, 42].

To study the effect of the isolated +19 in MDS we compared the MDS +19-*SRSF2* patients to an MDS-*SRSF2* cohort, lacking isolated +19. In addition, several MDS control groups were collected from the literature to corroborate our findings: non-selected MDS, MDS-RS, MDS-*SF3B1*, MDS-*SRSF2* and MDS +19.

Several features were unusual in MDS +19 and MDS/MPN +19 compared to MDS and MDS/MPN in general. Some of these (as discussed below) were also present in the MDS-*SRSF2* cohort, and thus may relate to the presence of *SRSF2* mutations, which is considered an early event in MDS pathogenesis [16], while other characteristics rather seemed to be related to the gain of an additional chromosome 19.

Aspects in MDS +19 and MDS/MPN +19 which were shared with MDS-*SRSF2* were the frequent presentation with anemia and the strong male predominance, both much more pronounced than in large series of unselected MDS and MDS/MPN patients, where less frequently anemia and only a slight male predominance were seen (55–70% male), especially MDS-*SF3B1* and MDS/MPN with RS showed only 38–50% male patients [5, 17, 41, 42].

The presence of erythroid hyperplasia and increased numbers of RS in 80% of the patients in both MDS +19 and MDS/MPN +19 was markedly different from the MDS-*SRSF2* cohort. Increased RS are reported in 5–40% of low risk MDS [5, 13, 49] and 18% of MDS/MPN patients [41], and are most frequently seen in MDS-*SF3B1* [18]. MDS-*SF3B1* is characterized by low blasts, increased RS, isolated erythroid dysplasia, slight male predominance and favorable prognosis [18]. The high correlation between *SF3B1* mutations and increased RS has resulted in the reclassification of the previous “MDS with ring sideroblasts” to “MDS with mutated *SF3B1*” (ICC [11]) and “MDS with low blast count and *SF3B1* mutation” (WHO5 [10]. So, according to these new classifications, the presence of increased numbers of RS is less important, although in the WHO “MDS with low blasts and RS” has been retained as an acceptable alternative to be used for cases with wildtype *SF3B1* and/or ≥ 15% ring sideroblasts.

A characteristic that seems to be related to the presence of +19 in MDS and MDS/MPN is the association of increased RS with *SRSF2* and *ASXL1* mutations. The presence of RS in +19 has been reported before in 3 out of 5 patients with CMML +19 [6] and single MDS +19 cases in the literature (see Table 2), further supporting the importance of +19.

In a study of 129 patients with MDS with > 5% RS, the majority (67%) had an *SF3B1* mutation, whereas a subgroup of 15 *SF3B1*-negative patients was reported to have an *SRSF2* mutation (11%) [43]. Detailed information on cytogenetics, for comparison with our cohort, was lacking and very few *ASXL1* mutations were reported in this study.

In MDS +19 patients, the disease course, with a 5-year survival rate of 30%, was not as indolent as previously described in MDS patients with an *SF3B1* mutation, with a 5-year survival rate of 80% [18]. It was characterized by disease progression, often with increased blasts, leuko- or monocytosis and/or fibrosis. Indeed, fibrosis in itself, which is seen in < 20% of MDS patients, is considered to be a risk factor in MDS [19, 50].

The role of +19 in disease evolution is unknown, but gene dosage of several genes located on chromosome 19, such as *CALR, CEBPA, MLL, EPOR* and *TCF3* could stimulate cell proliferation. Moreover, *TGFB1* and *IL-11* could induce fibrosis through stimulation of collagen synthesis by BM fibroblasts [51], and *DNMT1* may affect methylation [52].

Since *SRSF2* and *ASXL1* mutations on their own are not significantly associated with increased numbers of RS, the additional chromosome 19 might play an important role in this increase, although at the moment the mechanism remains unknown. A role of the cytosolic ferritin light chain gene *FTL* on chromosome 19 for the presence of RS seems unlikely, as RS are considered to result from an abnormal mitochondrial iron metabolism, with the gene for mitochondrial ferritin on chromosome 5 [53].

The order of the transforming events in MN +19 remains speculative. Based on comparison of MDS +19-*SRSF2* and MDS-*SRSF2*, we hypothesize that especially in elderly males the *SRSF2* and/or *ASXL1* mutation may be the initiating event, followed by or in combination with the acquisition of +19, which leads to the clinical presentation with erythroid hyperplasia, increased RS and fibrosis.

In conclusion, our study illustrates that the acquisition of trisomy 19, a rather unique cytogenetic event, influences clinical and biological features in MN patients. This results in an unusual combination that characterizes this group of patients, namely increased RS, predominantly male sex, *SF3B1* wildtype status, *SRSF2* or *ASXL1* mutations and fibrosis. The prognosis is less favorable than in MDS with low blasts and RS in the presence of *SF3B1* mutation, and warrants exclusion from this diagnostic category.

## Supplementary Information

Below is the link to the electronic supplementary material.

Supplementary file1 (PDF 444 KB)

## Data Availability

Data is provided within the manuscript or supplementary information files. Additional data are available from the corresponding author on reasonable request.
