# Supplementary material for: Analysis of myeloid neoplasms with isolated trisomy 19 reveals a novel MDS subgroup characterized by the presence of ring sideroblasts, fibrosis and SRSF2 and/or ASXL1 mutations
Source: J Hematop. 2025 Oct 9;18(1):45. doi: 10.1007/s12308-025-00659-1 (PMC12511170; doi:10.1007/s12308-025-00659-1)
Supplement: Supplementary file 1 — Supplementary file1 (PDF 444 KB) [file 12308_2025_659_MOESM1_ESM.pdf]

Journal of Hematopathology, Research article

Analysis of myeloid neoplasms with isolated trisomy 19 reveals a novel MDS subgroup characterized by the presence of ring sideroblasts, fibrosis and *SRSF2* and/or *ASXL1* mutations

Authors

Konnie M. Hebeda[1], Ludmila Boudová[2,3], Maarten F. Corsten[4], Nikola Ptáková [3,5], Torsten Haferlach[6], Aniek O. de Graaf[7], Jaroslav Cermak[8], Tomas Vanecek[2,3], Joop H. Jansen[7] , Marian J. P. L. Stevens-Kroef[9], Leonie I. Kroeze[1]

Affiliations

[1] Department of Pathology, Radboud University Medical Center, Nijmegen, Netherlands

[2] Department of Pathology, Charles University, Medical Faculty, Pilsen, Czech Republic

[3] Biopticka Laborator, Pilsen, Czech Republic

[4] Internal Medicine, Meander Medisch Centrum, Amersfoort, Netherlands

[5] Department of Biology and Medical Genetics, 2nd Faculty of Medicine and University Hospital Motol, Prague, Czech Republic

[6] Munich Leukemia Laboratory, München, Germany

[7] Laboratory Hematology, department of Laboratory Medicine, Radboud University Medical Center, Nijmegen, Netherlands

[8] Institute of Hematology and Blood Transfusion, MDS Registry, Prague, Czech Republic

[9] Department of Human Genetics, Radboud University Medical Center, Nijmegen, Netherlands

Corresponding Author:

Konnie M. Hebeda, konnie.hebeda@radboudumc.nl

Supplementary Table 1. Characteristics of patients with MN and isolated trisomy 19.

Patient	Sex age	Cytogenetics	Original diagnosis	ICC 2022	WHO 2022	*Mutated genes*	% RS	Anemia (A), Leucocytopenia (L), thrombocytopenia (T)	Fibrosis grade	Therapy	Last follow-up	Follow-up
MDS n=51												
1	M72	47,XY,+19[16]/46,XY[4]	MDS-RARS	MDS, NOS, SLD	MDS-LB-RS	[a] ***ASXL1 34%,*** *SETBP1 22%, U2AF1 43%*	>15	A	0		3 yrs	Alive , clonal evolution
2	M84	47,XY,+19[14]/46,XY[6]	MDS-MLD	MDS, NOS, MLD	MDS-LB-RS	[a] ***SRSF2 29%***	75	A	0		na	
3	M72	47,XY,+19[3]/46,XY[17]	MDS-RARS	MDS, NOS, SLD	MDS-LB-RS	[a] *RUNX1 31%*	27	A, L	1		2 yrs	Alive, lung carcinoma 1y later
4	M75	47,XY,+19[6]/46,XY[4]	MDS-RARS	MDS, NOS, SLD	MDS-LB-RS	[a] ***SRSF2 50%***	15-20	A, L, T	0		1 mo	Alive
5	M65	47,XY,+19[8]/46,XY[2]	MDS-RARS	MDS, NOS, SLD	MDS-LB-RS	[a] ***ASXL1 26% ,*** *SETBP1 11%, TP53 40%, U2AF1 34%*	>15	A	0/1 > 2	Transfusion, EPO	1 yr	Alive, progression to MDS-EB-F
6	F74	47,XX,+19[18]/46,XX[2]	MDS-RCMD-RS	MDS, NOS, MLD	MDS-LB-RS	[a] ***SRSF2 32%,*** *TET2 5%*	>15	na	0	ICT	3 mo	Alive, progression to AML with clonal evolution
7	M68	47,XY,+19[9]/46,XY[1]	MDS-NOS-MLD	MDS-NOS-MLD	MDS-LB-RS	***ASXL1 17%***	16	A, L	1	Transfusions, EPO, G-CSF, luspatercept	4 yrs	Alive
8	M81	47,XY,+19[7]/46,XY[3]	MDS-RCMD-RS	MDS, NOS, MLD	MDS-LB-RS	[a]***ASXL1 17%,*** *U2AF1 35%*	>15	A, T	1>2	Transfusions	2 yrs	Alive, progression to MDS-RAEB
9	F67	47,XX,+19[9]/46,XX[1]	MDS-MLD-RS	MDS, NOS, MLD	MDS-LB-RS	[a] ***SRSF2 46%,*** *IDH2 49%*	70	A	1 > 2	EPO, AZA ,luspatercept	2 yr	Alive, progression to MF2 and blast increase (CD117+), breast carcinoma
10	F63	47,XX,+19[22]	MDS-RARS	MDS, NOS, SLD	MDS-LB-RS	[a] ***SRSF2 52%***	>15	A, T	1	Transfusions, hydroxyurea	7.5 yrs	Progression CMML-like (monocytosis, splenomegaly) . Adenocarcinoma of colon. Died of acute brain hemorrhage
11	F75	47,XX,+19[5]/46,XX[15]	MDS-MLD-RS	MDS, NOS, MLD	MDS-LB-RS	[a]***ASXL1 33%,*** *KRAS 30%*	65	A, T	1	Transfusions, hydroxyurea	1 yr	Progression with MDS/MPN features Leucoytosis, splenomegaly). Died of infection
12	M80	47,XY,+19[13]/46,XY[7]	MDS RCMD-RS (WHO)	MDS, NOS, MLD	MDS-LB-RS	***SRSF2 42%, ASXL1 25%,*** *TET2*	26	na	na		na	
13	M58	47,XY,+19[13]/46,XY[7]	MDS RARS	MDS, NOS, SLD	MDS-LB-RS	***SRSF2 29%***	78	na	na		na	
14	M75	47,XY,+19[11]/46,XY[9]	MDS-RS	MDS, NOS	MDS-LB-RS	***SRSF2 38%,*** *TET2*	40	na	na		na	
15	M72	47,XY,+19[2]/46,XY[18]	MDS-RARS	MDS, NOS, SLD	MDS-LB-RS	[a] ***SRSF2 32%***	>15	A, L, basophilia	0	Supportive	2 yrs	1 yr: MDS-EB1, clonal evolution. Died of pneumonia
16	M85	47,XY,+19[16]/46,XY[4]	MDS-RS-MLD	MDS-SF3B1	MDS-LB-SF3B1	***SF3B1 24%***	30	na	na		na	
17	M82	47,XY,+19[13]/46,XY[8]	MDS-MLD	MDS, NOS, MLD	MDS-LB	***SRSF2 29%***	12	na	na		na	
18	M60	47,XY,+19[5]/46,XY[17]	MDS-RCMD	MDS, NOS, MLD	MDS-LB	[a]***ASXL1 25%,*** *U2AF1 35%*	<15	A, L, T	2	ICT, alloSCT	20 mo	Progression to MDS-RAEB2 and AML. Died of MOF
19	M75	47,XY,+19[17]/46,XY[3]	MDS-EB-1	MDS-EB	MDS-IB1	***SRSF2 41%,*** *KRAS*	74	na	na		na	
20	M80	47,XY,+19[16]/46,XY[5]	MDS-RAEB2	MDS/AML	MDS-IB2	[a] ***SRSF2 41%,*** *IDH2*	<15	na	1 > 2	Low dose Ara C AZA	6 yrs	Progression to MF2 and AML, clonal evolution. Died of pneumonia
21	M75	47,XY,+19[6]/46,XY[5]	MDS RAEB-2	MDS/AML	MDS-IB2	***ASXL1 15%,*** *RUNX1*	26	na	na		na	
22	M81	47,XY,+19[4]/46,XY[17]	MDS RAEB-2	MDS/AML	MDS-IB2	***ASXL1 38%,*** *TET2, BCOR, EZH2, KIT, RUNX1*	0	na	na		na	
23	M70	47,XY,+19[8]/46,XY[12]	MDS-EB-2	MDS/AML	MDS-IB2	***SRSF2 38%,*** *KIT, SETBP1*	11	na	na		< 1 yr	Progression to MDS/MPN, u
24	M67	47,XY,+19[4]/46,XY[16]	MDS-EB-2	MDS/AML	MDS-IB2	***ASXL1 27%,*** *MPL, RUNX1, SETBP1, U2AF1*	68	na	na		1 yr	Progression to AML, clonal evolution
25	F87	47,XX,+19[3]/46,XX,-7,+19[12]/48,XX,+19,+mar[2]/46,XX[3]	MDS-EB-MF	MDS-EB	MDS-F	[a] ***SRSF2 56%,*** *SETBP1 36%, DNMT3A 38%*	59	na	2		na	
26	M72	1)47,XY,+19[15]/46,XY[5] 2)47,XY,+19[5]/47,sl,i(14)(q10)[15] 3)47,XY,i(14)(q10),+19[3] 4) 47,XY,i(14)(q10),+19[20]	MDS-RCMD	MDS. NOS, MLD	MDS-LB	[a] ***SRSF2 40%,*** *TET2 40/40%*	Na	A, T	0		3 yrs	Alive, progression to AML
27	M72	47,XY,+19[4]/46,XY[6]	MDS-RCMD-MF	MDS, NOS, MLD	MDS-LB	[a] ***SRSF2, ASXL1,*** *EZH2*	na	A, L, T	3	Transfusions	3 mo	Alive
28	M55	47,XY,+19[10]	MDS-RCMD	MDS, NOS, MLD	MDS-LB	*U2AF1*	na	A, T	1>2		2 yr	Alive; progression CMML-like
29	M72	47,XY,+19[7]/46,XY[13]	MDS RAEB-2	MDS/AML	MDS-IB2	*TET2*	na	na	na		na	

30	M78	47,XY,+19[4]/46,XY[1]	MDS-EB2	MDS/AML	MDS-IB2	[a] ***ASXL1 33%***, *RUNX1 38%, U2AF1 43%, PHF6 6%*	na	A, T	0 > 2	Transfusions, EPO, AZA	3 yrs	Died with progressive fibrosis MF2
31	M75	47,XY,+19[3]/46,XY[7]	MDS-RCMD-RS	MDS, NOS, MLD	MDS-LB-RS	*na*	>15	A, L	na	Transfusions, EPO, AZA, DEC, LDAC	4 yrs	Progression to MDS-RAEB2 , died of AML
32	M83	47,XY,+19[11]/46,XY[9]	MDS-RS-MLD	MDS, NOS, MLD	MDS-LB-RS	*na*	20	A, L	na		na	
33	M91	47,XY,+19[5]/46,XY[1]	RCMD-RS	MDS, NOS, MLD	MDS-LB-RS	*na*	33	na	na		na	
34	M66	47,XY,+19[10]	MDS-MLD-RS	MDS, NOS, MLD	MDS-LB-RS	*na*	>15	A, T	na	Transfusions	1 yr	Died of pancreastic carcinoma
35	M82	47,XY,+19[5]/46,XY[15]	MDS-RS-MLD	MDS, NOS, MLD	MDS-LB-RS	*na*	32	na	na		na	
36	F69	47,XX,+19[19]/46,XX[1]	MDS RARS	MDS, NOS, SLD	MDS-LB-RS	*na*	>15	A, T	2 > 3		2 yrs	Alive, progression to MDS-RAEB2-MF3
37	M80	47,XY,+19[10]	MDS-RARS	MDS, NOS, SLD	MDS-LB-RS	*na*	>15	A	0 > 2		3 yrs	2 yrs: MDS-RAEB2-MF2, Died of T-ALL
38	M87	47,XY,+19[8]/46,XY[14]	t-MDS	MDS-EB, therapy-related	MDS-IB1	*na*	47	na	na		na	
39	M75	47,XY,+19[4]/46,XY[21]	MDS-EB-1	MDS-EB	MDS-IB1	*na*	0	na	na		na	
40	M64	47,XY,+19[20]	MDS RAEB2	MDS/AML	MDS-IB2	*na*	0	A, L, T	na	Transfusions, AZA, alloSCT	11 mo	Died of septic shock
41	M79	47,XY,+19[8]/46,XY[2]	MDS-RCMD	MDS. NOS, MLD	MDS-LB	*na*	na	A, L	0	Transfusions, EPO, AZA, lenalidomide	2 yrs	Died of progression to AML, clonal evolution
42	F89	47,XX,+19[11]/46,XY[1]	MDS-RCMD	MDS. NOS, MLD	MDS-LB	*na*	na	A	1	Transfusions, EPO	2 yrs,	Died of MDS
43	M78	47,XY,+19[10]	MDS-MLD	MDS, NOS, MLD	MDS-LB	*na*	na	A, L, T	na	Transfusions, EPO	<1 yr	Alive
44	M65	47,XY,+19[9]/46,XY[1]	MDS-MLD	MDS, NOS, MLD	MDS-LB	*na*	na	A	na	None	5 mo	Died, metastatic carcinoma
45	M78	47,XY,+19[10]	MDS-MLD	MDS, NOS, MLD	MDS-LB	*na*	na	A	1	Transfusions	1 yr	Alive
46	M85	47,XY,+19[10]	MDS-MLD	MDS, NOS, MLD	MDS-LB	*na*	na	A	0		na	
47	M83	47,XY,+19[9]/46,XY[1]	MDS-RCMD	MDS, NOS, MLD	MDS-LB	[a]*failed*	na	A	1 > 2		4 mo	Alive with oesophageal carcinoma, progression to MF2, clonal evolution
48	F90	47,XX,+19[15]/48,XX,+19,+mar[3]/46,XX[2]	MDS-RAEB-1	MDS-EB	MDS-IB1	[a]*failed*	na	A, L, T	1		na	
49	F78	47,XX,+19[7]/46,XX[10]	MDS-EB2	MDS/AML	MDS-IB2	*na*	na	A, L	1		2 yrs	Alive
50	M73	47,XY,+19[21]/46,XY[1]	MDS RAEB-2	MDS/AML	MDS-IB2	*na*	na	na	na		na	
51	M70	47,XY,+19[10]	MDS-EB-F	MDS-EB	MDS-F	[a] *failed*	na	A	2		4 yrs	Died of progression to AML
MDS/MPN n=11												
52	M70	47,XY,+19[10]/46,XY[10]	MDS/MPN, U	MDS/MPN, NOS	MDS/MPN-NOS	***SRSF2 37%, ASXL1 6/28% SF3B1 38%,*** *PPM1D*	74	na	na		na	
53	M53	47,XY,+19[17]/46,XY[3]	MDS/MPN, U	MDS/MPN, NOS	MDS/MPN-NOS	***SRSF2 28%,*** *ASXL2*	89	na	na		na	
54	M84	47,XY,+19[19]/46,XY[1]	MDS/MPN, U	MDS/MPN, NOS	MDS/MPN-NOS	*na*	35	na	na		na	
55	M61	47,XY,+19[24]	MDS/MPN, U	MDS/MPN, NOS	MDS/MPN-NOS	[a] ***SRSF2 38%,*** *TET2 48%*	40	A	1		na	
56[b]	M52	47,XY,+ 19	MDS/MPN, U	MDS/MPN, NOS	MDS/MPN-NOS	[a] ***SRSF2 45%,*** *TET2 45%, NPM1 10%*	<15	A, T, leucocytosis	1\> 2	Transfusions, 2x alloSCT	3 yrs	Relapse with AML and progressive MF2, clonal evolution. Died of infection
57	M76	47,XY,+19[7]/46,XY[8]	CMML	CMML	CMML-1	[a] ***SRSF2***	na	T, monocytosis	1 > 2		3 yrs	Alive, progressive fibrosis MF2, clonal evolution
58	M81	47,XY,+19[2]/46,XY[30]	aCML	aCML	MDS/MPN w neutrophilia	[a] ***SRSF2 51%,*** *SETBP1 49%*	na	thrombocytosis, leucocytosis	1		2 yrs	Alive
59	M72	47,XY,+19[2]/46,XY[14]	MDS/MPN, U	MDS/MPN, NOS	MDS/MPN-NOS	***ASXL1 50%***	na	na	na		na	
60	M73	arr(19)x3[0.5] [trisomy 19]	SM-AHN-CMML	SM-AMN-CMML	SM-AHN-CMML	***SRSF2 47%, ASXL1 30%,*** *KIT 7%, JAK2 2%*	na	A, T, monocytosis	2		1 mo	Died from progression
61	M81	47,XY,+19[10]/46,XY[2]	CMML	CMML	CMML-1	[a] *failed*	na	A, leukocytosis	0	Supportive	na	
62	M79	47,XY,+19[3]/46,XY[19]	CMML	CMML	CMML	*na*	na	na	1		na	
Excluded AML n=10												
63	M65	47,XY,+19[3]/46,XY[27]	AML ex MDS	AML-MR	AML-MR	[a] ***ASXL1 28%,*** *SETBP1 42%, U2AF1 38%, JAK2*	0	na	0	ICT	5 mo	Relapse MDS-RAEB1
64	F53	47,XX,+19[10]	AML-M5	AML-NPM1	AML-NPM1	*NPM1*	na	na	na	2x ICT, autoSCT	4 yrs	Alive, remission
65	F29	47,XX,+19[4]/46,XX[6]	AML	AML-NOS	AML	[a] *KDM6A*	na	leucocytosis	1	ICT + 2x alloSCT	2 yrs	Alive
66	M67	47,XY,+19[16]/46,XY[4]	AML M1	AML, NOS		*TET2, CEBPA*	na	na			na	
67	M75	47,XY,+19[2]/46,XY[19]	AML M2	AML-MR	AML-MR	*RUNX1, ZRSR2*	na	na			na	
68	M74	47,XY,+19[2]/46,XY[4]	AML	AML-MR	AML-MR	***ASXL1****, EZH2, RUNX1*	na	na			na	

69	M47	47,XY,+19[10]/ 46,XY[20]	APL	APL	APL	*na*	na	na		AlloSCT	4 yrs	Alive
70	M21	47,XY,+19[14],	AML-M1	AML, NOS	AML	*na*	na	na		ICT+autoSCT	6 yrs	Alive, relapse acute leukemia
71	F4 mo	47,XX,+19[8]/4 6,XX[2],	AML-M0	AML, NOS	AML minimal differenti ation	*na*	na	A		Transfusions, alloSCT	2 yrs	Died of relapse AML. Clonal evolution
72	M70	47,XY,+19[4]/4 6,XY[2]	AML ex MDS/MPN	AML, NOS, ex MDS/MPN, NOS	AML	*na*	na	na			na	
Exluded patients other reasons n=24												
73	M62	Initial karyotype normal, at progression 47,XY,+19[13]/ 46,XY[6]	MDS-RA	MDS, NOS, SLD	MDS-LB	*na*	no	A		ICT, alloSCT	10 yrs	Progression to RAEB2. Died of relapse +19 at disease progression
74	M81	Initial karyotype unknown, at progression 47,XY,+19[9]/4 6,XY[13]	MDS-RCMD	MDS, NOS, MLD	MDS-LB	[a]***ASXL1 30%,*** *RUNX1 5%, TET2 28%, ZRSR2 14%*	0	A, T, monocytosis	0>3	Supportive	4 yrs	Progression +19 at disease progression
75	M70	47,XY,+19[1]/4 6,XY[19]	AML ex MDS	AML-MR	AML-MR	***ASXL1*** *2/5%*	na	na				Only 1 cell with +19
76	M77	47,XY,+19[7]/4 6,XY[13]	Possibly MDS			*na*	0	na			na	No definitive diagnosis
77	M70	47,XY,+19[5]/4 6,XY[17]	Possibly MDS			*na*	4	na			na	No definitive diagnosis
78	M73	47,XY,+19[12]/ 46,XY[8]	Possibly MDS			***SRSF2 29%,*** *TET2*	6	na			na	No definitive diagnosis
79	M?	MDS normal karyotype, 47,XY,+19[14]/ 46,XY[6]	AML ex MDS			*na*	na	na			na	+19 at disease progression
80	F74	MDS normal karyotype, 47,XX,+19[9]/4 6,XX[1]	AML-M2, ex MDS	AML-MR	AML-MR	*na*	na	A, T, leucocytosis		Transfusions, ICT	3 yrs	MDS-RAEB relapse, died +19 at disease progression
81	F75	47,XX,+19[7]/4 6,XX[4]	AML, ex MDS	AML-MR	AML-MR	[a] ***SRSF2,*** *IDH1, RUNX1*	na	A, L		ICT, AZA	1 yr	Died of relapse AML +19 at disease progression
82	M61	MDS normal karyotype, 47,XY,+19[22]	MDS/MPN, U	MDS/MPN, U	MDS/MP N,U	[a] ***SRSF2, ASXL1 31%,*** *IDH2, NRAS, SLC6A6:: RUNX1T1*	na	A	1	Supportive	27 mo	Died of progression +19 at disease progression
83	M67	47,XY,+19[3]/4 6,XY[19]	CMML/AM L?			***SRSF2 51%***	na	na	1		na	No definitive diagnosis
84	M69	47,XY,+19[13]/ 46,XY[7]	MN, possibly CMML			***SRSF2 57%,*** *TET2*	5	na			na	No definitive diagnosis
85	M?	47,XY,+19[11]/ 46,XY[8]	MDS			*na*	na	na	na		na	No definitive diagnosis
86	M?	47,XY,+19[20]	MDS			*na*	na	na	na		na	No definitive diagnosis
87	M?	47,XY,+19[16]/ 46,XY[4]	MDS			*na*	na	na	na		na	No definitive diagnosis
88	M?	47,XY,+19[9]/4 6,XY[1]	MDS			*na*	na	na	na		na	No definitive diagnosis
89	M?	47,XY,+19[2]/4 6,XY[18]	Unspecified myeloid neoplasm			*na*	na	na	na		na	No definitive diagnosis
90	F76	47,XX,+19[10]	tMDS-F, Post MTX	MDS, therapy-related		*na*	na	A, L, T	2	Transfusions, EPO. hydrea	3 yrs	Died of progression No definitive diagnosis
91	M?	47,XY,+19[10]	MN, post MTX	MDS, therapy-related/MDS		*na*	na	na		ICT, alloSCT	5 yrs	<1yr AML. Died of brain tumor No definitive diagnosis
92	F81	47,XX,+19[21]	MDS/MPN?			*na*	na	na	2		na	No definitive diagnosis
93	M86	47,XY,+19[25]	MN			*na*	na	na	na		na	No definitive diagnosis
94	F75	47,XX,+19[3]/4 7,X,t(X;7)(p22; p1?5),+19[5]/46 ,XX[2]	MN			*na*	na	na	na		na	No definitive diagnosis
95	F85	47,XX,+19 [7]/46,XX [3]	MDS			*na*	na	na			na	Lacking data
96	M	47,XY,+19[2]/4 6,XY[31]	CMML	CMML	CMML	*na*	na	na	na		na	**Lacking data**
97	M	47,XY,+19[11]/ 46,XY[10]	CMML	CMML	CMML	*na*	na	na	na		na	**Lacking data**

alloSCT= allogeneic stem cell transplantation; arr= SNP array; autoSCT= autologous stem cell transplantation; AZA= azacytidine; ICT= intensive chemotherapy; F= female; M= male; MF= significant myelofibrosis (grade 2 or 3), if known with grade; mo= months; na= data not available; RS= ring sideroblasts; SM= systemic mastocytosis; yrs= years

[a] analyzed on DNA extracted from the bone marrow biopsy

[b] Patient described in: Hebeda et al (2021) Progression, transformation, and unusual manifestations of myelodysplastic syndromes and myelodysplastic-myeloproliferative neoplasms: lessons learned from the XIV European Bone Marrow Working Group Course 2019. Annals of Hematology 100:117-133. https://doi.org/10.1007/s00277-020-04307-9

Supplementary Table 2. Gene panels of next-generation sequencing (NGS) performed on the paraffin embedded bone marrow biopsies in the Biopticka Laborator, Pilsen, Czech Republic and Radboudumc, Nijmegen, Netherlands.

Gene	Biopticka	Radboud
ABL1	X	
ANKRD26	X	
ASXL1	X	X
ASXL2		
BCOR	X	X
BCORL1		
BRAF	X	X
CALR	X	X
CBL	X	X
CEBPA	X	
CSF3R	X	X
CSNK1A1	X	
DDX41	X	X
DNMT3A	X	X
ETNK1	X	X
ETV6	X	X
EZH2	X	X
FLT3	X	X
GATA1	X	
GATA2	X	X
IDH1	X	X
IDH2	X	X
JAK2	X	X
KIT	X	X
KRAS	X	X
MPL	X	X
NF1		
NFE2		X
NOTCH1		
NPM1	X	X
NRAS	X	X
PHF6	X	X
PIGA		
PPM1D		X
PTPN11	X	X
RAD21	X	
RUNX1	X	X
SETBP1	X	X
SF3B1	X	X
SRSF2	X	X
STAG2	X	X
TET2	X	X
TP53	X	X
U2AF1	X	X
UBA1		X
WT1	X	X
ZRSR2	X	X

Supplementary Figure 1. Kaplan-Meier curve of survival for the MDS patients with isolated trisomy 19 (MDS+19).

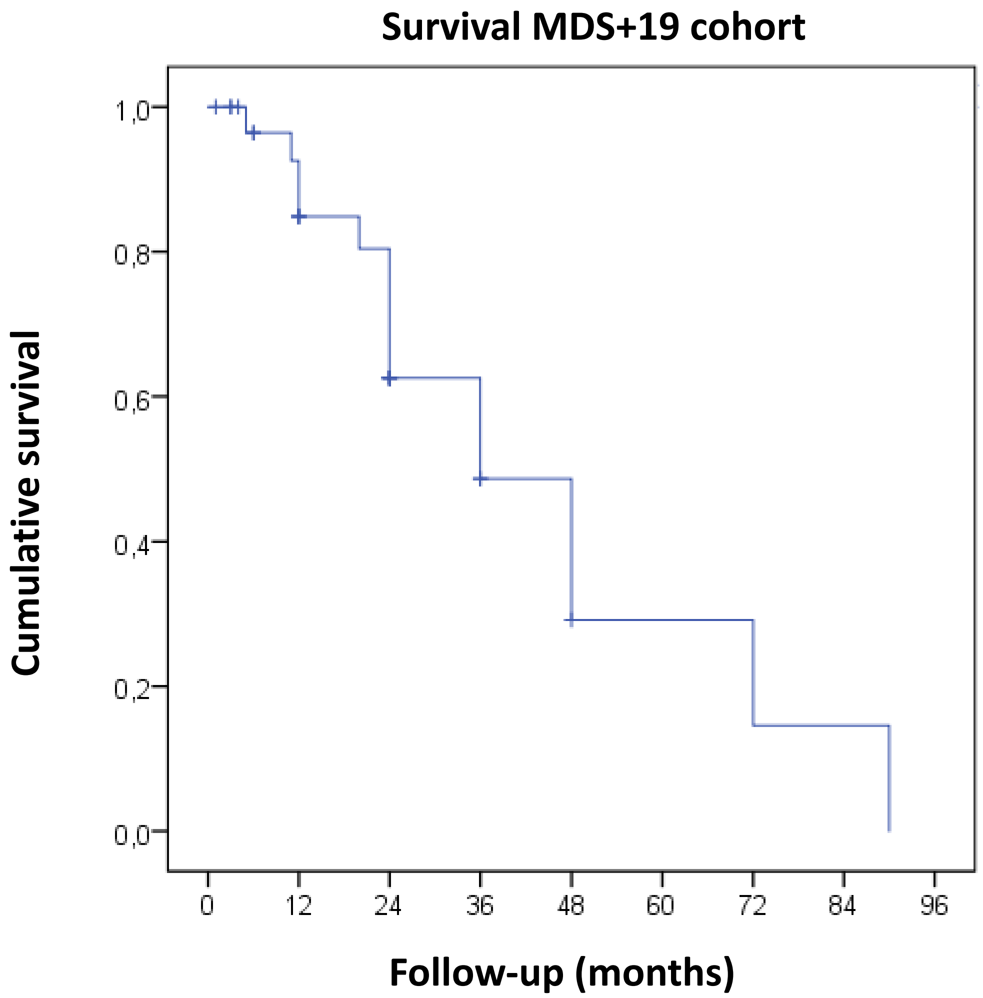

Pnts 31 19 9 5 2 2 1 1
